# Bax expression measured by AQUAnalysis is an independent prognostic marker in oral squamous cell carcinoma

**DOI:** 10.1186/1471-2407-12-332

**Published:** 2012-08-01

**Authors:** Pinaki Bose, Alexander C Klimowicz, Elizabeth Kornaga, Stephanie K Petrillo, T Wayne Matthews, Shamir Chandarana, Anthony M Magliocco, Nigel T Brockton, Joseph C Dort

**Affiliations:** 1 Department of Oncology, University of Calgary, Calgary, Canada; 2 Department of Oncology, University of Calgary, Calgary, Canada; 3 Division of Otolaryngology-Head and Neck Surgery, University of Calgary, Calgary, Canada; 4 Department of Population Health Research, Alberta Health Services – Cancer Care, Calgary, Canada; 5 Present Address: H. Lee Moffitt Cancer Center & Research Institute, 12902 Magnolia Drive, Tampa, FL, 33612, USA

**Keywords:** AQUA, Bax, Bcl-2, Bcl-XL, Oral cancer, prognosis

## Abstract

**Background:**

Resistance to apoptosis is a hallmark of cancer and proteins regulating apoptosis have been proposed as prognostic markers in several malignancies. However, the prognostic impact of apoptotic markers has not been consistently demonstrated in oral squamous cell carcinoma (OSCC). This inconsistency in reported associations between apoptotic proteins and prognosis can be partly attributed to the intrinsic low resolution and misclassification associated with manual, semi-quantitative methods of biomarker expression measurement. The aim of this study was to examine the association between apoptosis-regulating proteins and clinical outcomes in oral squamous cell carcinoma (OSCC) using the quantitative fluorescence immunohistochemistry (IHC) based AQUAnalysis technique.

**Methods:**

Sixty-nine OSCC patients diagnosed between 1998–2005 in Calgary, Alberta, Canada were included in the study. Clinical data were obtained from the Alberta Cancer Registry and chart review. Tissue microarrays (TMAs) were assembled from triplicate cores of formalin-fixed paraffin embedded pre-treatment tumour tissue. Bax, Bcl-2 and Bcl-XL protein expression was quantified using fluorescent IHC and AQUA technology in normal oral cavity squamous epithelium (OCSE) and OSCC tumour samples. Survival was analyzed using Kaplan-Meier plots and the Cox proportional hazard model.

**Results:**

Bax expression was predominantly nuclear in OCSE and almost exclusively cytoplasmic in OSCC. No similar differences in localization were observed for Bcl-2 or Bcl-XL. Only Bax expression associated with disease-specific survival (DSS), with 5-year survival estimates of 85.7% for high Bax versus 50.3% for low Bax (p = 0.006), in univariate analysis. High Bax expression was also significantly associated with elevated Ki67 expression, indicating that increased proliferation might lead to an improved response to radiotherapy in patients with elevated Bax expression. In multivariate analyses, Bax protein expression remained an independent predictor of DSS in OSCC [HR 0.241 (0.078-0.745), p = 0.013].

**Conclusions:**

The AQUA technique used in our study eliminates observer bias and provides reliable and reproducible estimates for biomarker expression. AQUA also provides essential measures of quality control that cannot be achieved with manual biomarker scoring techniques. Our results support the use of Bax protein expression as a prognostic marker in conjunction with other clinico-pathological variables when designing personalized treatment strategies for OSCC patients.

## Background

Oral cavity squamous cell carcinoma (OSCC) is the most common form of head and neck squamous cell carcinoma (HNSCC). The annual estimated incidence of oral cancer is almost 300,000 worldwide [1]. OSCC is characterized by significant morbidity and mortality and presents a considerable challenge to clinical management due to its high propensity of loco-regional recurrence and cervical lymph node dissemination. Conventional multimodal therapy for OSCC is associated with significant toxicity and functional impairment. Despite major advances in diagnostic imaging, surgical reconstruction and delivery of radiation therapy (RT) and chemotherapy, the average 5-year survival for OSCC remains close to 50% [2].

Although several clinical and histopathological and molecular markers have been proposed [3,4], current clinical care is directed, primarily, by the tumour-node-metastasis (TNM) classification system. The TNM system describes the attributes of the cancer in terms of the size and extension of the primary tumour, its nodal involvement and presence of distant metastasis. However, TMN staging is of limited prognostic value in individual patients since it does not consider the underlying biology of tumour cells. The biological mechanisms that determine the course of OSCC are poorly understood. The human-papilloma virus (HPV) is a well-known prognostic marker in certain HNSCC subsites such as the orpharynx, where more than 70% of cases have been reported to be HPV-positive [5-7]. However, a similar association between HPV and OSCC has not been established [8]. Therefore, novel prognostic and predictive biomarkers are required to direct optimal management of OSCC.

Apoptotic pathways are critical cell-autonomous tumour surveillance mechanisms that guard against the development of neoplasms. Indeed, evading apoptosis is considered one of the hallmarks of cancer [9,10]. The role of mediators of apoptosis has been investigated in the development and progression of several cancers [11], including OSCC [12-15]. The evolutionarily conserved B cell lymphoma-2 (Bcl-2) family of proteins controls apoptosis by regulating the permeability of the mitochondrial outer membrane [16,17]. Members of the Bcl-2 family are functionally classified as either anti-apoptotic or pro-apoptotic. Most cells express a variety of Bcl-2 family proteins and their balance dictates cell fate [18]. Bcl-2-associated X protein (Bax) [19], is a pro-apoptotic member of the Bcl-2 family. Bax promotes mitochondrial outer membrane permeabilization, a hallmark of apoptosis, by forming homo-oligomers on the mitochondrial membrane [20]. Two prominent anti-apoptotic members of the Bcl-2 family are Bcl-2 and Bcl-2-related gene long isoform (Bcl-XL) [21]. These anti-apoptotic proteins preserve mitochondrial membrane integrity by directly inhibiting the pro-apoptotic Bcl-2 family members.

Since Bcl-2 and Bcl-XL are anti-apoptotic proteins, they are expected to function as oncogenes in cancer cells and pro-apoptotic proteins such as Bax are expected to act as tumour-suppressors. According to these assumptions, cancers over-expressing Bcl-2 or Bcl-XL should have worse prognosis, while tumours over-expressing Bax should be associated with better clinical outcomes. However, studies analyzing the prognostic significance of apoptotic proteins in OSCC have yielded contradictory results. Camisasca *et al*. [22] reported that the elevated expression of Bax, Bcl-2 and Bcl-XL was associated with better overall survival, disease-specific survival (DSS) and lower recurrence rates in OSCC. Yuen *et al*. [23] observed no association between Bcl-2 expression and survival in OSCC. Another study reported associations between increased Bcl-2 expression, neck metastasis and poor survival, but did not detect any prognostic significance for Bax expression [24]. Other studies have reported improved prognosis in OSCC patients whose tumours express low levels of Bcl-2 and high levels of Bax [25,26]. All these studies have used immunohistochemistry to assess the expression of the three apoptotic markers, however, no previous studies have used automated quantitative fluorescent immunohistochemistry [27] to assess the expression levels of apoptotic proteins in OSCC. Automated quantitative analysis (AQUA) of tissue microarrays (TMAs) can be used to measure protein expression, specifically within tumour cells, on a continuous scale. The technology serves to remove observer bias and provides essential standards for quality control that are not available in conventional immunochemistry analysis techniques involving manual examination of individual TMA cores. In the present study, we evaluated the expression of Bcl-2, Bcl-XL and Bax using the AQUA technique in 69 OSCC tumour samples and compared these results to normal oral cavity squamous epithelium (OCSE). We also examined the association of apoptotic biomarker expression with clinico-pathological variables and DSS in OSCC.

## Methods

### Cohort identification

**Figure 1:**
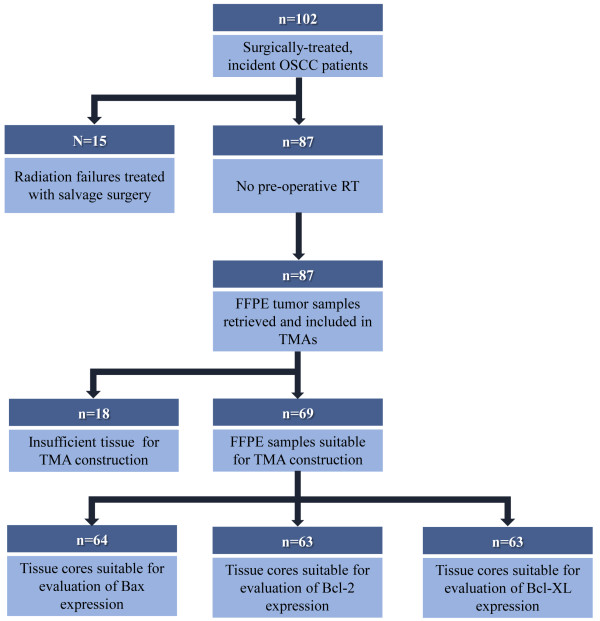
**Consort Diagram outlining patient selection criteria for the OSCC study cohort.** Sufficient tumour tissue for TMA construction was available for 69 patients. TMA cores from 64 patients were included in the analysis of Bax expression; TMA cores from 63 patients were analyzed for Bcl-2 and Bcl-XL expression. The same 63 patients were consistent between Bax, Bcl-2 and Bcl-XL expression analyses. The loss of 5 patients (Bax expression analysis) and 6 patients (Bcl-2 and Bcl-XL expression analysis) was due to the insufficient tumour, core loss during sectioning, or folding of the tissue in all three cores of a particular patient.

Institutional ethics approval was obtained from Conjoint Health Research Ethics Board at the University of Calgary, in accordance with the Tri-Council Policy Statement on Research with Human Subjects. The study cohort consisted of 102 patients with OSCC who were treated with primary surgery (resection and neck dissection) between 1998 and 2005 at the Foothills Medical Centre, Calgary, Alberta, Canada (Figure 1). The inclusion criteria for patients were the histological confirmation of OSCC with no prior history or treatment for head and neck cancer. Patients received post-operative radiation therapy in any of 3 situations: 1) metastatic cancer extending beyond the lymph node capsule 2) multiple metastatic lymph nodes 3) positive surgical margins. Patient demographics and clinical outcome were collected by a combination of comprehensive chart review and data retrieval from the Alberta Cancer Registry.

### Tissue microarray (TMA) construction

Surgically resected, treatment naïve formalin-fixed, paraffin–embedded (FFPE) tumour samples were available for 69 of the 102 patients included in the study and were retrieved for TMA construction (Figure 1). Hematoxylin and eosin stained slides obtained from surgical specimens were reviewed by the study pathologist (A.M.) to confirm the diagnosis. FFPE tumour blocks with sufficient tumour to obtain three 0.6 mm cores were selected for each patient and marked for sampling and inclusion into TMAs. TMAs were assembled from triplicate 0.6 mm cores randomly sampled from the tumour-containing area of each FFPE block using a Beecher Manual Tissue Microarrayer (Beecher Instruments Inc. Sun Prairie, WI). Five samples of normal OCSE, separate from the tumour containing regions of archived FFPE tissue blocks, were also included in the TMAs as reference samples to establish normal biomarker expression levels.

### Quantitative fluorescent immunohistochemistry

We used the HistoRx^TM^ AQUA platform and fluorescent immunohistochemistry [27] to quantify the expression of Bcl-2, Bcl-XL, Bax and Ki67 in the tumour compartment of each TMA core. TMA sections (4 μm) were deparaffinized in xylene, rinsed in ethanol, and rehydrated. Heat induced epitope retrieval for Ki67 was performed by heating slides to 121 °C in a citrate-based Target Retrieval Solution (pH 6.0) (Dako) for 6 minutes in a decloaking chamber (Biocare Medical). For Bcl-2, Bcl-XL and Bax, an EDTA-containing Target Retrieval Solution (pH 9.0) (Dako) was used for 3 minutes. TMA slides were stained for Bcl-2 (rabbit monoclonal anti-Bcl-2, 1:1000 dilution, Epitomics), Bcl-XL (rabbit monoclonal anti-Bcl-XL, 1:500 dilution, Cell Signaling), Bax (rabbit monoclonal anti-Bax, 1:5000 dilution, Epitomics) or Ki67 (mouse monoclonal anti-Ki67, 1:5000 dilution, Dako). Rabbit Envision + or mouse Envision + (Dako) was used in conjunction with tyramide-Cy3 (Perkin-Elmer) for Ki67 or tyramide-Cy5 (Perkin-Elmer) for the Bcl-2 family members to visualize the expression level of each biomarker. The epithelial (tumour) compartment was identified by staining with a guinea-pig anti-pan-cytokeratin (PCK) antibody (ACRIS) and an Alexa488 conjugated anti-guinea pig secondary antibody. Slides were mounted using Prolong Gold Anti-fade with DAPI (Invitrogen). Slides were scanned by HistoRx PM-2000™ and analyzed by AQUAnalysis® software (version 2.2.1.7). The tumour compartment was defined as the PCK-positive area for each TMA core, the tumour nuclear compartment was defined as the DAPI-positive area within the tumour compartment, and the Ki67 compartment area was defined as the Ki67 positive area within the tumour nuclear compartment. Unusable areas within each image (such as those with folded tissue) where manually cropped. Also, cores with insufficient tumour area (<20% tumour) were excluded from further analysis. AQUAnalysis® software was used to calculate AQUA scores representing the exposure time-adjusted pixel intensity density of biomarker proteins within the tumour compartment area of each core. Ki67 expression was reported as the percentage of tumour nuclear area that was positive for Ki67. Expression of a given biomarker was also determined in normal OCSE.

### Statistical Analysis

The distribution of AQUA scores was assessed with respect to normal oral cavity squamous epithelium to determine the relative expression level of each biomarker in tumour tissue. The median tumour AQUA score for each biomarker was selected to dichotomize patients into low or high expression groups for each biomarker. A biomarker was defined to be overexpressed in tumour tissue if the median AQUA score in the tumour exceeded the median AQUA score in normal OCSE. The Fisher's exact test was used to compare patient characteristics between the two patient groups defined by low/high Bax, Bcl-2 and Bcl-XL expression. Kaplan-Meier survival analysis was used to assess 5-year DSS. Survival was measured from the date of diagnosis to the date of death or the date of last follow-up. Cox proportional hazards analyses were conducted to assess the impact of clinical covariates. Pathological T-stage and N-stage were included in the multivariate analysis because of their clinical significance and their significant association with DSS in univariate analysis (Table 1). The stage covariate was not included in the Cox model because it is collinear with T-stage and N-stage. All statistical analyses were performed using Stata 11 data analysis and statistical software (StataCorp LP, Tx, USA).

**Table 1:** Association of demographic and clinico-pathological characteristics with expression levels of Bax, Bcl-2 and Bcl-XL

	**N=69**		**Univariate Analysis**			**Bax**^**a**^		**n=64**	**Bcl-2**^**a**^		**n=63**	**Bcl-XL**^**a**^		**n=63**
	**# OF CASES**	**(%)**	Hazard Ratio	95% CI	p-value	Low	High	p-value	Low	High	p-value	Low	High	p-value
**GENDER**			1.72	0.693 - 4.286	0.24			0.419			0.109			0.419
Male	49	71.01				20	24		18	25		20	23	
Female	20	28.99				12	8		13	7		12	8	
**AGE**^**b**^			1.35	0.543 - 3.364	0.52			1.000			0.315			0.616
<60 years	29	42.03				15	14		12	17		16	13	
≥60 years	40	57.97				17	18		19	15		16	18	
**pT STATUS**			2.55	1.022 - 6.357	0.05			0.616			0.207			0.616
1 + 2	38	55.07				19	16		20	15		19	16	
3 + 4	31	44.93				13	16		11	17		13	15	
**pN STATUS**			7.470	2.467 - 22.611	<0.0001			0.315			0.802			1.000
N0	39	56.52				15	20		16	18		17	17	
N1 + N2	30	43.48				17	12		15	14		15	14	
**SMOKING HISTORY**			0.65	0.245 - 1.702	0.38			0.075			0.148			0.237
Never	15	21.74				11	4		10	5		10	5	
Ever	54	78.26				21	28		21	27		22	26	
**ALCOHOL HISTORY**			0.46	0.162 - 1.282	0.14			1.000			1.000			1.000
Never	11	17.46				5			4	4	5		5	4	
Ever	52	82.54				27	23		23	26		24	24		
**TUMOUR DIFFERENTIATION**			1.62	0.772 - 3.427	0.2			1.000			0.923			0.525	
Well	14	22.95				7	6		6	7		8	5		
Moderate	38	62.30				18	18		19	16		15	20		
Poor	9	14.75				4	3		4	3		3	4		
**TREATMENT**			2.16	0.629 - 7.412	0.22			0.257			0.011			0.164	
Surgery	18	26.09				6	11		13	4		6	11		
Surgery + RXT	51	73.91				26	21		18	28		26	20		
**STAGE**			4.68	1.358 - 16.087	0.01			1.000			0.797			1.000	
I + II	26	37.68				11	12		12	11		12	11		
III + IV	43	62.32				21	20		19	21		20	20		
**PROLIFERATION (Ki67)**			0.6	0.231 - 1.539	0.29			0.001			0.617			0.211	
Low	32	50.00				23	9		17	15		19	13		
High	32	50.00				9	23		14	17		13	18		

^a^Cut-point selected at median.

^b^Median value.

## Results

### Cohort characteristics

Our study, adheres to the REMARK criteria [28]. Sixty-nine patients met the inclusion criteria for the study and had FFPE tissue available for TMA construction. Median follow-up time for the cohort was 44 months. Univariate analyses of potentially relevant clinico-pathological characteristics are presented in Table 1. Stratified analyses according to low/high Bax, Bcl-2 and Bcl-XL are also presented in Table 1. The study cohort consisted of 49 males and 20 females (median age 62.6 years, range 25.7-83.3 years). Our cohort contained both low (pT1/pT2; n = 38) and high (pT3/pT4; n = 31) pathologic T-stages. Nodal involvement (pN1/pN2) was present in 30 patients. Twenty six of the 69 patients were classified as clinical stage I/II, and 43 were classified as clinical stage III/IV. Treatment consisted of surgery alone or surgery followed by RT for 18 and 51 patients, respectively. The 5 year disease-specific survival for the cohort was 72%.

### Bax protein expression

**Figure 2:**
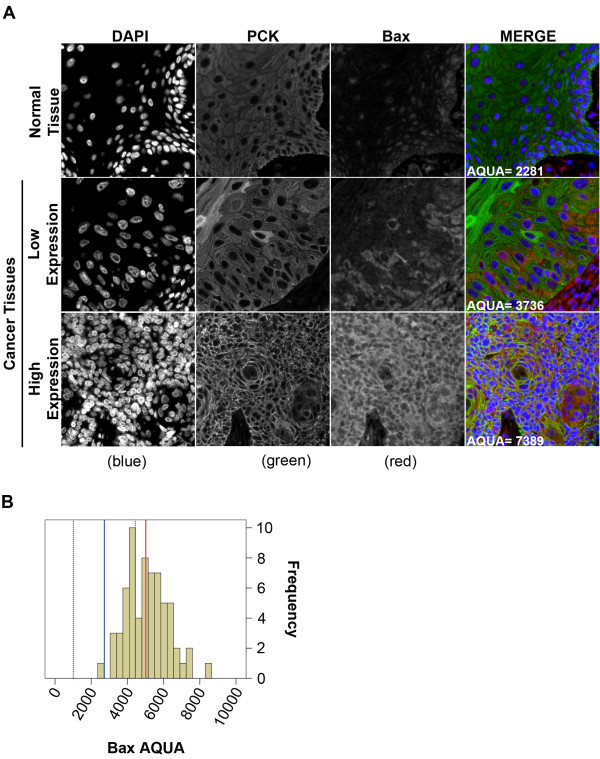
**Fluorescent immuno-histochemical staining of Bax using the HistoRx.**^**TM **^**AQUA platform.** (**A**) Representative examples of quantitative fluorescent IHC images for Bax expression in normal OCSE (top panel) and OSCC (two bottom panels). AQUA scores represent the expression level of Bax within the pan-cytokeratin defined epithelial/tumour compartment. DAPI-stained nuclei are depicted in blue, pan-cytokeratin-stained epithelial/tumour cells are depicted in green, and Bax protein expression is depicted in red. (**B**) Histogram distribution representing Bax expression within OSCC tumour samples. The solid blue line represents median Bax expression in normal OCSE, the broken blue lines represent +/−2 standard deviations from median Bax expression in normal OCSE, and the solid red line represents median Bax expression in OSCC.

We observed weak, predominantly nuclear expression of Bax in OCSE; expression was most prominent in the basal epithelial layer. In contrast, Bax expression in OSCC samples was distinctly cytoplasmic and ranged from sporadic expression in low expressing tumours to ubiquitous expression in high expressing tumours (Figure 2A). The median Bax AQUA score in normal OCSE was 2650 [95% C.I. 937–4362] (Figure 2B, broken blue lines). Median Bax expression in the OSCC tumour samples was 5015 [95% C.I. 4458–5483]; this exceeded the 95% C.I. of Bax expression in normal OCSE and consequently, met our definition of over-expression. Bax over-expression within the tumour was significantly associated with a high proliferation index (p = 0.001) determined by greater than median nuclear Ki67 expression (Table 1).

### Bcl-2 protein expression

**Figure 3:**
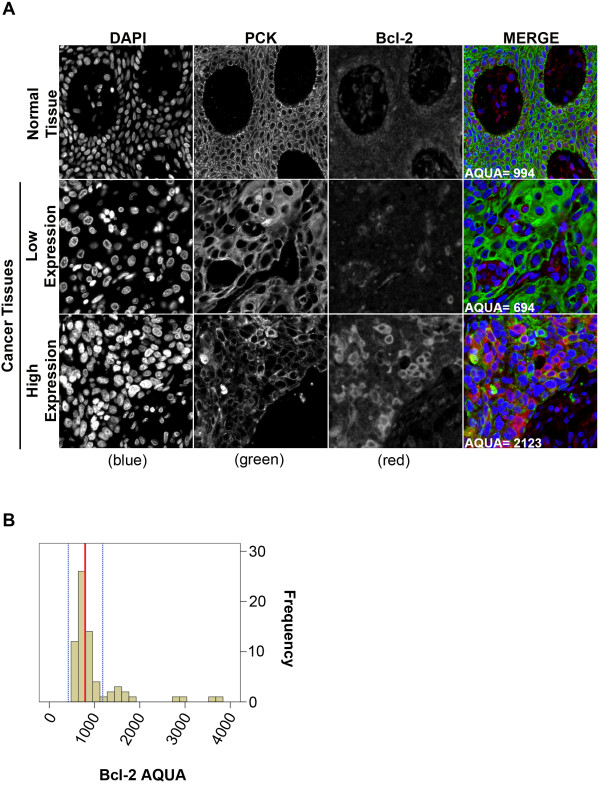
**Fluorescent immuno-histochemical staining of Bcl-2 using the HistoRx**^**TM **^**AQUA platform:** (**A**) Representative examples of quantitative fluorescent IHC images for Bcl-2 expression in normal OCSE (top panel) and OSCC (two bottom panels). AQUA scores represent the expression level of Bcl-2 within the pan-cytokeratin defined epithelial/tumour compartment. DAPI-stained nuclei are depicted in blue, pan-cytokeratin-stained epithelial/tumour cells are depicted in green, and Bcl-2 protein expression is depicted in red. (**B**) Histogram distribution representing Bcl-2 expression within OSCC tumour samples. The solid blue line represents median Bcl-2 expression in normal OCSE, the broken blue lines represent +/−2 standard deviations from median Bcl-2 expression in normal OCSE, and the solid red line represents median Bcl-2 expression in OSCC.

The typical Bcl-2 staining pattern in normal OCSE was weakly cytoplasmic with a slightly increased level of expression in the basal epithelial layer (Figure 3A). Bcl-2 expression in OSCC samples was also cytoplasmic and ranged from diffuse expression in low-expressing tumours to strong sporadic expression in high-expressing tumours (Figure 3A). The median Bcl-2 AQUA score in normal OCSE was 807 [95% C.I. 427–1187] (Figure 3B, broken blue lines). Median Bcl-2 expression in OSCC was 783 [95% C.I. 718–830], which overlapped with the median expression of Bcl-2 in OCSE (Figure 3B). Significantly more patients with high Bcl-2 expressing tumours (greater than median) were treated with RT in addition to surgery (p = 0.011) (Table 1).

### Bcl-XL protein expression

**Figure 4:**
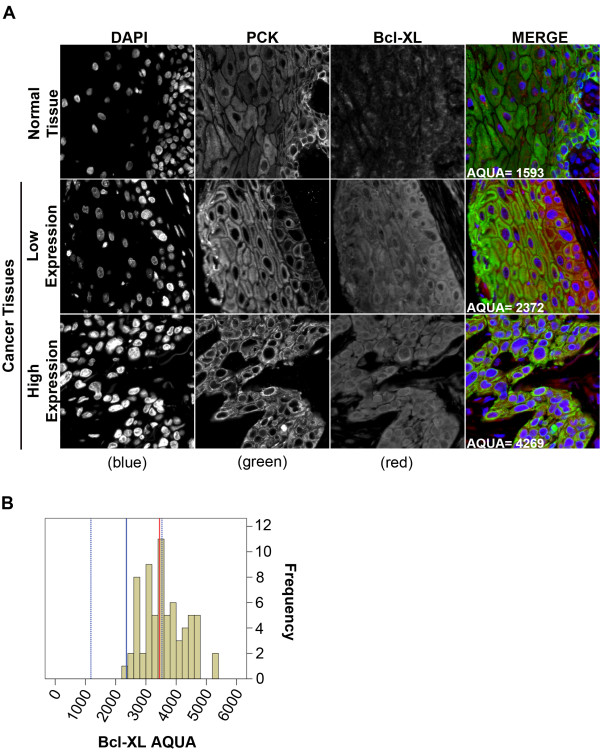
**Fluorescent immuno-histochemical staining of Bcl-XL using the HistoRx**^**TM **^**AQUA platform.** (**A**) Representative examples of quantitative fluorescent IHC images for Bcl-XL expression on normal OCSE (top panel) and OSCC (two bottom panels). AQUA scores represent the expression level of Bcl-XL within the pan-cytokeratin defined epithelial/tumour compartment. DAPI-stained nuclei are depicted in blue, pan-cytokeratin-stained epithelial/tumour cells are depicted in green, and Bcl-XL protein expression is depicted in red. (**B**) Histogram distribution representing Bcl-XL expression within OSCC tumour samples. The solid blue line represents median Bcl-XL expression in normal OCSE, the broken blue lines represent +/−2 standard deviations from median Bcl-XL expression in normal OCSE, and the solid red line represents median Bcl-XL expression in OSCC.

Both nuclear and cytoplasmic Bcl-XL staining was observed in normal OCSE, with consistently stronger staining in the cytoplasm. Bcl-XL was strongly expressed in all OSCC samples and, similar to OCSE, exhibited a strong cytoplasmic and weak nuclear staining pattern (Figure 4A). The median Bcl-XL AQUA score in normal OCSE was 2480 [95% C.I. 1305–3655] (Figure 4B, broken blue lines). Median Bcl-XL expression in the OSCC tumour samples was 3450 [95% C.I. 3248–3705] which approached the upper limit of Bcl-XL expression in normal OCSE 95% C.I.; we defined this as borderline overexpression. High Bcl-XL expression was not associated with any clinico-pathologic variables (Table 1).

### Prognostic impact of apoptosis-regulating proteins

**Figure 5:**
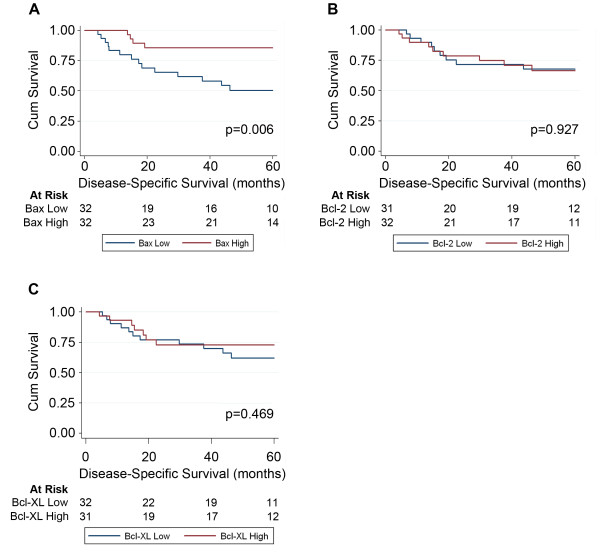
**Kaplan-Meier survival analysis of Bax, Bcl-2 and Bcl-XL expression.** Kaplan-Meier survival curves and corresponding risk tables for 5 year disease-specific survival in OSCC patients with below median or above median (**A**) Bax, (**B**) Bcl-2, and (**C**) Bcl-XL.

High Bax expression was associated with significantly improved 5-year DSS (Figure 5A). The survival estimates for patients with high and low Bax-expressing tumours were 85.7% and 50.3%, respectively. High Bax expression was also associated with a high proliferation index (as measured by greater than median Ki67 expression) (p = 0.001, Table 1). Although not statistically significant, we observed a positive correlation between Ki67 expression and DSS ( Additional file1: Figure S1). Neither Bcl-2 nor Bcl-XL expression levels were associated with DSS (Figures 5B and 5 C). Since the Bcl-2/Bax ratio and the Bcl-XL/Bax ratio has been shown to associate with survival outcomes in OSCC and other cancers [26], we examined if a similar association existed in our OSCC cohort using automated quantitative IHC analysis. We did not observe a significant association between these ratios and DSS in our study ( Additional file 2: Figure S2).

A Cox proportional hazards model was used to assess the independent prognostic value of Bax, the only biomarker that reached statistical significance in univariate analysis. Lymph node status (negative/positive) and pathologic T-stage (T1/T2 vs. T3/T4) were included with Bax (high/low) in the multivariate model since these variables are routinely used by physicians when making treatment decisions. All three variables, Bax [HR 0.236 (0.076-0.731), p = 0.012], lymph node status [HR 7.344 (2.090-25.803), p = 0.002] and pathologic T-stage [HR 3.261 (1.234-8.618), p = 0.017], were significantly and independently associated with DSS (Table 2).

**Table 2:** Univariate and multivariate (Cox proportional hazards) analysis of 5-year disease-specific survival

	**Univariate**		**Multivariate**	
	**Hazard Ratio (95% C.I.)**	**p-value**	**Hazard Ratio (95% C.I.)**	**p-value**
**Bax**	0.237 (0.078 - 0.721)	0.005	0.236 (0.076 - 0.731)	0.012
**pT Status**	2.549 (1.022 - 6.357)	0.043	3.261 (1.234 - 8.618)	0.017
**pN Status**	7.470 (2.467 - 22.611)	<0.001	7.344 (2.090 - 25.803)	0.002

Pathological T-stage and N-stage were included in the multivariate analysis based on their acknowledged clinical significance in OSCC and their significant association with DSS in univariate analysis.

## Discussion

The current prediction of prognosis in OSCC is based primarily on the TNM staging system and does not consider the contribution of molecular biomarkers or underlying tumour biology when assigning stage or predicting outcomes. This may be attributed to the lack of robust and reliable techniques for measuring biomarker expression that will help validate their use in the clinic. Several studies have investigated whether changes in the expression levels of apoptosis-associated proteins are associated with survival outcomes in OSCC [14,22,29,30]. However, a lack of consensus in these studies has precluded the use of apoptotic protein expression as a biomarker in the clinic. Automated quantitative IHC-based techniques, such as AQUA, can be used to eliminate observer bias in the measurement of protein expression. Furthermore, AQUA can measure protein expression within distinct sub-cellular and tissue compartments (e.g. defining expression in the nucleus/cytoplasm and tumour/stroma), possibly improving the clinical applicability of this technique. In the present study, we measured the expression of three apoptosis-regulating proteins using AQUA and found that Bax expression was an independent prognostic marker in OSCC.

Several reports suggest that Bax plays a tumour suppressor role in human malignancies and high Bax expression is associated with favourable prognosis in several cancer types [31-35]. Our univariate analysis showed that high Bax expression is associated with improved prognosis. Furthermore, multivariate analysis using Cox proportional hazards regression to adjust for important clinical covariates, confirmed that Bax expression was independently associated with DSS in OSCC. Tumours with high Bax expression are likely to be more susceptible to apoptosis than their low Bax-expressing counterparts. Previous studies have reported that Bax expression is selectively induced in apoptosis-proficient cells [36]; therefore, OSCC cells with elevated Bax expression could be those that are primed for apoptosis due to the inherent genetic instability associated with malignant transformation.

High Bax expression was associated with increased Ki67 expression, a marker of proliferation. This is consistent with previously reported associations between Bax expression and proliferation in several cancer sites [37,38]. Bax over-expression increases cellular proliferation and accelerates G1 to S-phase transition [39,40]; this increased cellular proliferation in genetically unstable cancer cells can lead to mitotic catastrophe, thereby augmenting cell death induced by apoptosis in response to radiotherapy [41]. The increased proliferation associated with elevated Bax expression could confer an improved response to radiotherapy in patients with high Bax. Indeed, stratification by radiation treatment demonstrated that the association between Bax expression and DSS was most pronounced in patients that received adjuvant radiotherapy ( Additional file 3: Figure S3); however, we were unable to appropriately assess this effect in our cohort. Although not statistically significant, a weak association was observed between high Ki67 expression and improved 5-year DSS ( Additional file 1: Figure S1).

Since the increased expression of Bcl-2 and Bcl-XL proteins are integral to the survival of malignant cells, it was not surprising that we did not observe any association between the expression of these proteins and 5-year DSS. In effect most tumours rely on the increased expression of antiapoptotic proteins and therefore these proteins will not likely identify unique patient subsets with regard to outcome. We also investigated if the balance between pro-apoptotic and anti-apoptotic proteins was associated with DSS by testing the impact of Bcl-2/Bax and Bcl-XL/Bax ratios on survival outcome. However, we did not find any significant association between these ratios and 5-year DSS. A possible explanation might be the altered expression of additional apoptosis-regulating factors that determine cell fate [30].

We evaluated the cellular localization of the three apoptosis-associated proteins in our study and found that Bax expression was predominantly nuclear in normal OCSE but primarily cytoplasmic in OSCC. Previous studies have also reported the presence of Bax and Bcl-2 in the nucleus [42-44], however the function of nuclear Bax in our study remains unclear. The sub-cellular localization of Bcl-2 and Bcl-XL was similar in normal and tumour tissue. Bax was the only biomarker that exhibited a difference in sub-cellular localization between normal OCSE and OSCC and was also the only apoptotic protein significantly associated with prognosis. The translocation of Bax from the nucleus to the cytoplasm in OSCC is consistent with increased Bax function at the mitochondria, leading to improved sensitivity to radiotherapy-induced apoptosis in tumours with elevated Bax expression.

Bax and Bcl-XL were over-expressed in most tumour samples, while a majority of the tumour samples expressed Bcl-2 at levels equivalent to normal OCSE. These results suggest that Bcl-XL might be the major anti-apoptotic protein in oral carcinogenesis and the increased Bax expression may be a compensatory response to elevated Bcl-XL levels. Interestingly, we observed that patients treated with surgery alone were over-represented in the low Bcl-2 group, whereas patients additionally treated with post-surgery RT were more common in the high Bcl-2 group (Table 1). However, since Bcl-2 did not correlate with treatment outcome, this observation may be attributed to chance rather than a true biologic phenomenon.

A major strength of our study is the use of automated quantitative immunohistochemistry to quantify biomarker expression. The high-throughput technique eliminates observer bias and provides reliable and reproducible estimates for biomarker expression. We used normal tissue samples to obtain physiologically relevant estimates of biomarker expression and define over-expression or down-regulation of apoptotic biomarker in our OSCC cohort. Our study included samples from a single head and neck sub-site (oral cavity) and a uniformly treated cohort from a single cancer center, thus minimizing treatment heterogeneity and increasing the reliability of our results.

Our study is limited by its relatively small sample size and retrospective nature. Despite the advantages of the AQUA technique for discovery-phase studies, it is not widely available in routine laboratory diagnostics at present, limiting its current clinical utility. However, the robustness and reliability of AQUA makes it amenable for adoption as a routine tool for prognostication in the imminent future.

## Conclusions

High Bax expression could serve to stratify OSCC patients with better prognosis, in a manner similar to HPV status in cancers of the oropharynx. Since both chemotherapy and radiotherapy work by inducing apoptosis in rapidly proliferating cells, our results suggest that combined modality treatments should be explored in patients with low Bax and Ki67-expressing tumours [45]. Confirmation of the prognostic significance of Bax expression in OSCC in prospective studies could translate into a simple immuno-histochemical test that might be useful for treatment selection and prognostication. Such an approach could lead to personalized approaches in treating this challenging disease.

## Abbreviations

AQUA: Automated Quantitative Analysis; Bax: Bcl-2-associated X protein; Bcl-2: B-cell lymphoma 2; Bcl-XL: Bcl-2-related gene long isoform; DAPI: 4',6-diamidino-2-phenylindole; DSS: Disease-Specific Survival; FFPE: Formalin-Fixed, Paraffin–Embedded; HNSCC: Head and Neck Squamous Cell Carcinoma; HPV: Human-Papilloma Virus; IHC: Immunohistochemistry; OCSE: Oral Cavity Squamous Epithelium; OSCC: Oral Squamous Cell Carcinoma; TMA: Tissue Microarray; TNM: Tumour-Node-Metastasis.

## Competing interests

This study was funded by the Ohlson Research Initiative, which functions within the Faculty of Medicine at the University of Calgary. The corresponding author (JCD) is the Director of the Ohlson Research Initiative. This does not alter our adherence to all BMC Cancer policies as detailed in the guide for authors. All other authors declare no conflict of interest.

## Authors' contributions

PB designed and coordinated the study, contributed to data analysis and interpretation and drafted the manuscript. ACK contributed to study design, performed survival analysis and helped with manuscript preparation. EK performed statistical analyses and edited the manuscript. SKP performed the fluorescence IHC and AQUA. TWM and SC contributed patients to the study, reviewed the manuscript and were involved in the interpretation of the clinical data. AMM was the designated study pathologist and performed the review of H&E stained slides to confirm diagnosis and select areas of tumour for TMA preparation. NTB and JCD conceived the study and helped with manuscript preparation and editing. JCD contributed to study design, clinical cohort assembly, data analysis and interpretation. All authors read and approved the final manuscript.

## Authors' information

PB is a Postdoctoral research fellow with the Ohlson Research Inititative in Head and neck cancer, University of Calgary. ACK is an adjunct assistant professor and the Director of the Functional Tissue Imaging Unit (FTIU) at the Tom Baker Cancer Centre, University of Calgary. EK is a research technician at the FTIU. SKP is a research technician at the FTIU. TWM is an otolaryngologist and head and neck surgeon. He is also the Division Chief of Otolaryngology, Head and Neck Surgery in the Department of Surgery, University of Calgary. SC is an otolaryngologist and head and neck surgeon in the Division of Otolaryngology - Head and Neck Surgery, Department of Surgery, University of Calgary. AMM is presently the Chief of Pathology at H. Lee Moffitt Cancer Center & Research Institute. NTB is a molecular cancer epidemiologist and research scientist with Population Health Research, Alberta Health Services – Cancer Care. JCD is an otolaryngologist, head and neck surgeon and epidemiologist. He is also the Director of the Ohlson Research Inititative in Head and Neck Cancer.

## Pre-publication history

The pre-publication history for this paper can be accessed here:

http://www.biomedcentral.com/1471-2407/12/332/prepub

## Supplementary Material

Additional file 1 ** Figure S1. Kaplan-Meier survival analysis of Ki67 expression.** Kaplan-Meier survival curves and corresponding risk tables for 5 year disease-specific survival in OSCC patients with below median or above median Ki67. Click here for file

Additional file 2 ** Figure S2. Kaplan-Meier survival analysis of ratios between pro-apoptotic and anti-apoptotic Bcl-2 family proteins.** Kaplan-Meier survival curves and corresponding risk tables for 5 year disease-specific survival in OSCC patients with below median or above median (A) Bcl-2/Bax and (B) Bcl-XL/Bax. Cut-points were selected at median. Click here for file

Additional file 3 ** Figure S3. Kaplan-Meier survival analysis of Bax expression with respect to treatment in OSCC.** Kaplan-Meier curves for 5-year disease-specific survival using (A) Bax expression for patients treated with surgery and post-operative radiation and (B) Bax expression for patients treated with surgery only (no radiation). Cut-points were selected at median. Click here for file
