# Supplementary figures and images for: Bax expression measured by AQUAnalysis is an independent prognostic marker in oral squamous cell carcinoma
Source: BMC Cancer. 2012 Aug 1;12:332. doi: 10.1186/1471-2407-12-332 (PMC3487960; doi:10.1186/1471-2407-12-332)

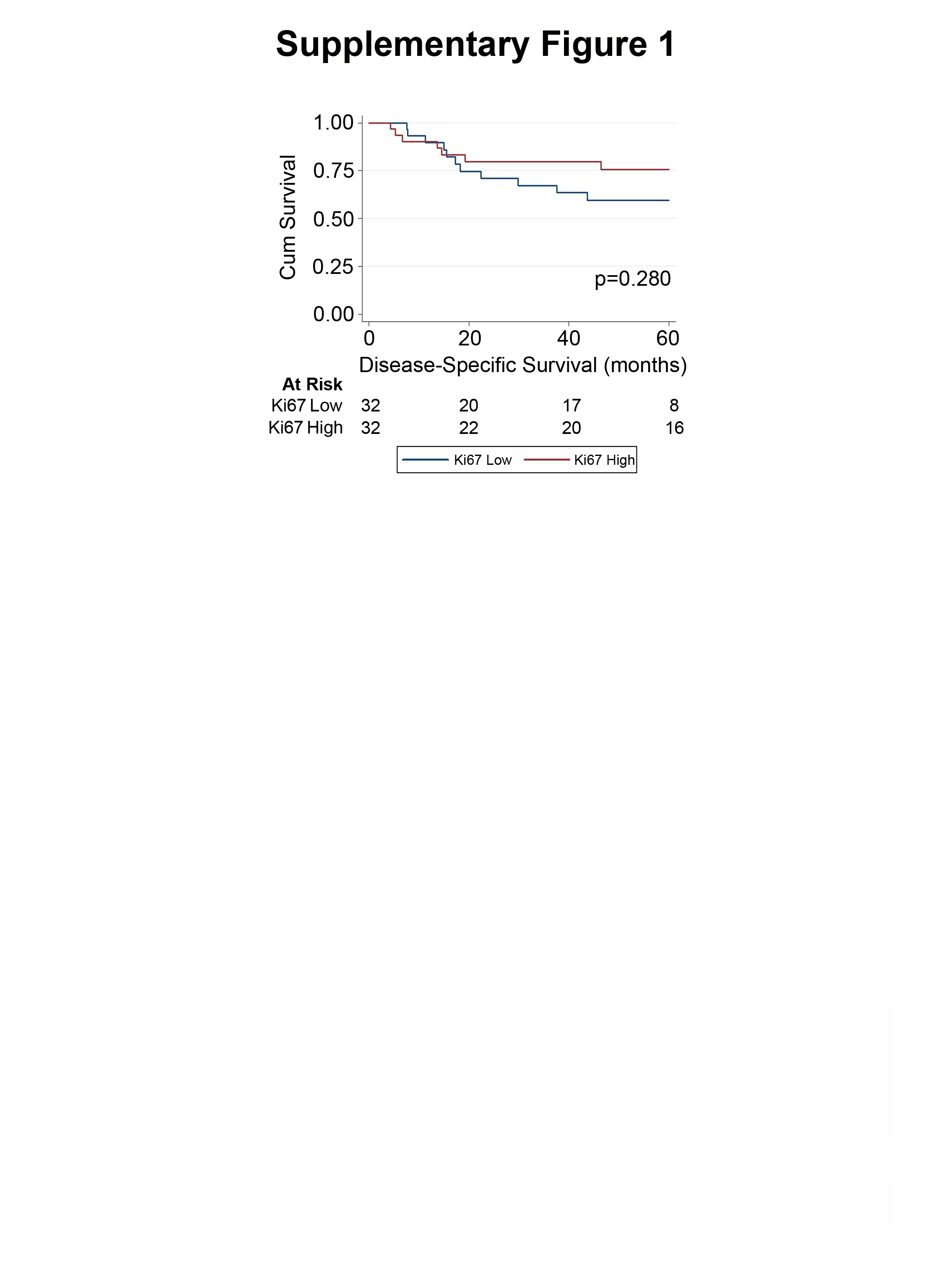

Supplement: Additional file 1 — Figure S1. Kaplan-Meier survival analysis of Ki67 expression. Kaplan-Meier survival curves and corresponding risk tables for 5 year disease-specific survival in OSCC patients with below median or above median Ki67. [file 1471-2407-12-332-S1.tiff]

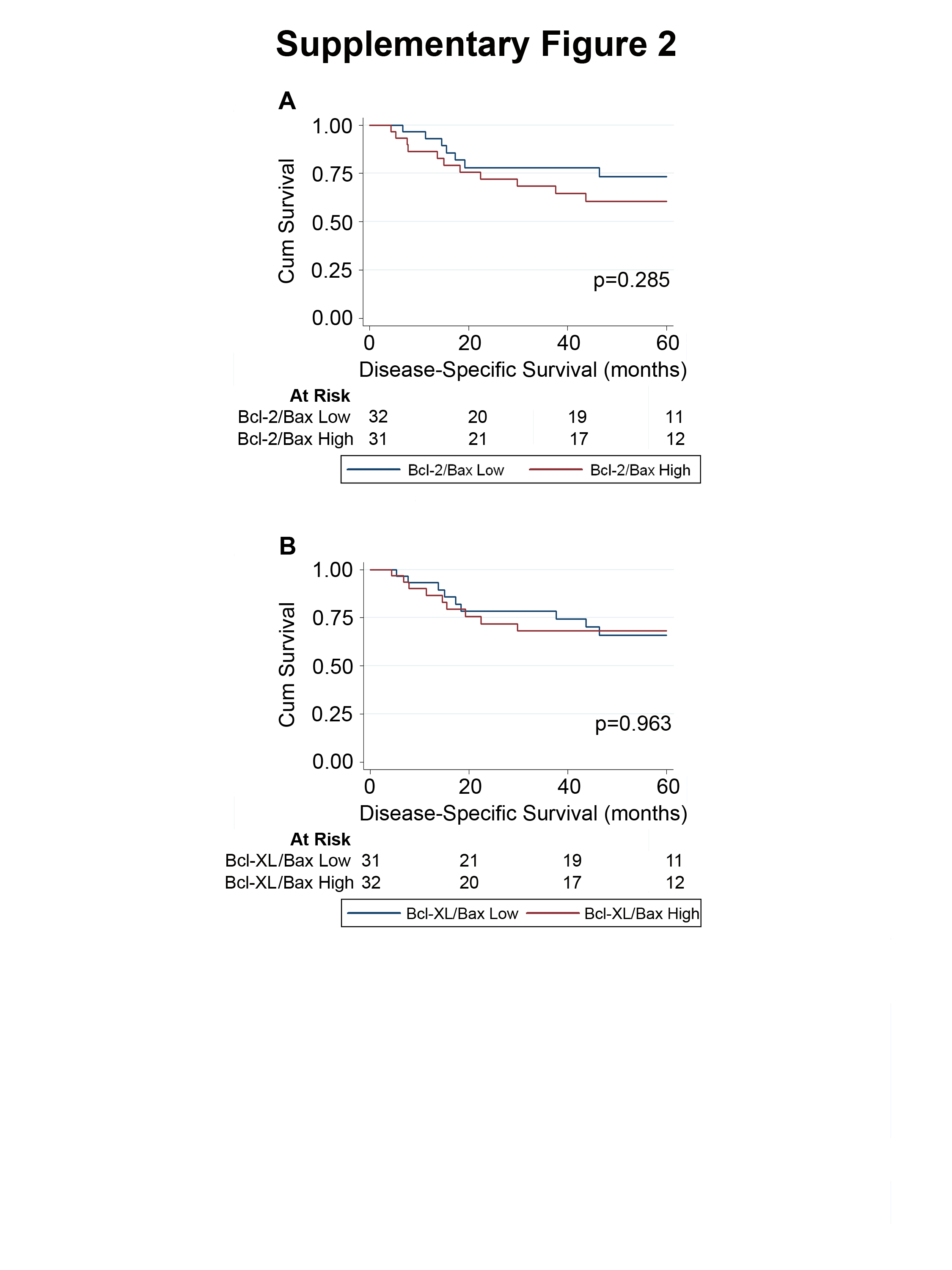

Supplement: Additional file 2 — Figure S2. Kaplan-Meier survival analysis of ratios between pro-apoptotic and anti-apoptotic Bcl-2 family proteins. Kaplan-Meier survival curves and corresponding risk tables for 5 year disease-specific survival in OSCC patients with below median or above median (A) Bcl-2/Bax and (B) Bcl-XL/Bax. Cut-points were selected at median. [file 1471-2407-12-332-S2.tiff]

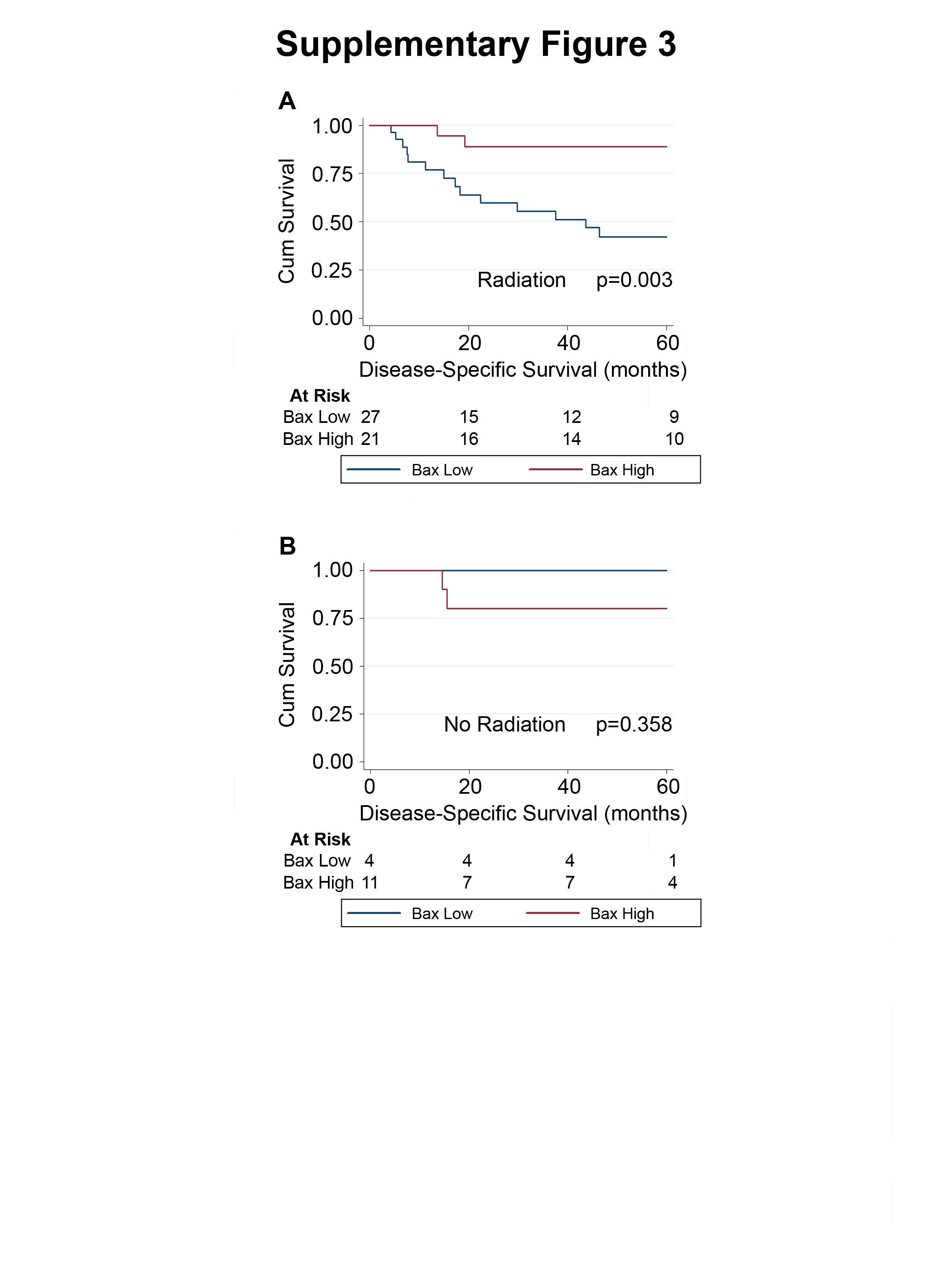

Supplement: Additional file 3 — Figure S3. Kaplan-Meier survival analysis of Bax expression with respect to treatment in OSCC. Kaplan-Meier curves for 5-year disease-specific survival using (A) Bax expression for patients treated with surgery and post-operative radiation and (B) Bax expression for patients treated with surgery only (no radiation). Cut-points were selected at median. [file 1471-2407-12-332-S3.tiff]
